# Alix: A Candidate Serum Biomarker of Alzheimer’s Disease

**DOI:** 10.3389/fnagi.2021.669612

**Published:** 2021-06-15

**Authors:** Yingni Sun, Jin Hua, Gen Chen, Jianjie Li, Jiateng Yang, Hongwei Gao

**Affiliations:** ^1^School of Life Sciences, Ludong University, Yantai, China; ^2^Department of Pharmacology, Institute of Materia Medica, Chinese Academy of Medical Sciences and Peking Union Medical College, Beijing, China; ^3^Department of Anesthesiology, Kunhua Hospital, The First People’s Hospital of Yunnan Province, Kunming, China; ^4^Department of Hepatobiliary Surgery III, Guizhou Provincial People’s Hospital, Guiyang, China

**Keywords:** sensitivity, specificity, vascular dementia, diagnosis, ROC analysis

## Abstract

Alzheimer’s disease (AD) is the most common fatal neurodegenerative disease of the elderly worldwide. The identification of AD biomarkers will allow for earlier diagnosis and thus earlier intervention. The aim of this study was to find such biomarkers. It was observed that the expression of Alix was significantly decreased in brain tissues and serum samples from AD patients compared to the controls. A significant correlation between Alix levels and cognitive decline was observed (*r* = 0.80; *p* < 0.001) as well as a significant negative correlation between Alix and Aβ_40_ in serum levels (*r* =−0.60, *p* < 0.001). The receiver operating characteristic curve (ROC) analysis showed the area under the curve (AUC) of Alix was 0.80, and the optimal cut-off point of 199.5 pg/ml was selected with the highest sum of sensitivity and specificity. The diagnostic accuracy for serum Alix was 74%, with 76% sensitivity and 71% specificity respectively, which could differentiate AD from controls. In addition, the expression of Alix was found to be significantly decreased in AD compared to vascular dementia (VaD). ROC analysis between AD and VaD showed that the AUC was 0.777, which could be indicative of the role of serum Alix as a biomarker in the differential diagnosis between AD and VaD. Most surprisingly, the decreased expression of Alix was attenuated after the treatment of Memantine in different AD animal models. In conclusion, our results indicate the possibility of serum Alix as a novel and non-invasive biomarker for AD for the first time.

## Introduction

Alzheimer’s disease (AD) is one of the most common progressive neurodegenerative diseases in the elderly, accounting for 60–80% of all cases (Sabayan and Sorond, 2017; Garre-Olmo, 2018). AD is characterized by cognitive impairment with the progressive loss of basal forebrain cholinergic neurons, deposition of extracellular senile plaques formed by amyloid β (Aβ), and intracellular neurofibrillary tangles (NFTs) of hyperphosphorylated tau (Scheltens et al., 2016). Although many recent pieces of research have revealed a great deal about AD (Hodson, 2018; Jack et al., 2018), the exact pathogenesis is not yet fully known. Current treatments can only help improve the clinical symptoms, but cannot delay or reverse the progression of AD. Thus, earlier diagnosis will allow for the earlier intervention of the therapeutic strategies that might have the best efficacy. The identification of AD biomarkers would have great values to aid in the diagnosis of AD. However, to date, there is no non-invasive and cost-effective biomarker to improve the diagnosis.

Until now, the only definitive way to diagnose AD has been to search for plaque with a brain autopsy after the patient dies (DeTure and Dickson, 2019). The acknowledged biomarkers in cerebrospinal fluid (CSF) include Aβ, total tau (T-tau), and phosphorylated tau (P-tau; Olsson et al., 2016), but we found it difficult to distinguish AD from controls due to the nonspecific changes in AD. Moreover, obtaining CSF through the invasive lumbar puncture on large numbers of elderly individuals is challenging. All these limit the application of CSF biomarkers. In recent years, many scientists focused on blood biomarkers (Olsson et al., 2016; Hampel et al., 2018; Penner et al., 2019; Zetterberg and Burnham, 2019). Many candidate proteins in blood have been found by the proteomic approach (Kitamura et al., 2017; Shen et al., 2017; Petersen et al., 2020), but the following validation showed unsatisfactory results. It is necessary to continue to look for viable diagnostic biomarkers for AD.

Alix is also called ALG-2 interacting protein X, and it participated in a regulatory Ca^2+^-dependent pathway through interaction with ALG-2 protein (Missotten et al., 1999). ALG-2, an EF-hand calcium-binding protein, could regulate the cell death program underlying apoptosis by changing the Ca^2+^ concentration following endoplasmic reticulum stress (Maki et al., 2016; Mercier et al., 2016). Numerous observations suggest that the brains from AD display an early impairment in the endosomal system, which appears in neurons long before amyloid plaque and neurofibrillary tangle formation (Nixon, 2005). It has been recently demonstrated that Alix and ALG-2 form a molecular coupling between endosomes and neuronal death in the presence of calcium, apical caspases, and tumor necrosis factor α receptor 1 (Mahul-Mellier et al., 2009). It was reported that Alix is also involved in caspase 9 activation and apoptosis triggered by calcium (Strappazzon et al., 2010). Given that, we speculated whether Alix could act as a valuable biomarker in AD. Thus, we detect the levels of Alix from serum and brain tissues in AD patients and healthy controls, and we also evaluated the diagnostic values of Alix as an AD biomarker. It was very meaningful for the discovery of ideal biomarkers of AD.

## Materials and Methods

### Control, AD, and VaD Brains

We collected frozen cortical and hippocampal tissues of 12 AD patients, eight vascular dementia (VaD) patients, and 12 age-matched controls. They were matched with the control group in terms of age of onset, gender, body mass index (BMI), and educational level. Written informed consent regarding the donation was provided and then approved by the Institutional Review Board (IRB). The National Institute of Neurological and Communicative Disorders and Stroke and the Alzheimer’s Disease and Related Disorders Association (NINCDS-ADRDA) Workgroup criteria were set for the clinical diagnosis of AD, and the same was organized for the National Institute for Neurological Disorders and Stroke and Association Internationale pour la Recherche et I’ Enseignement en Neurosciences (NINDS-AIREN) criteria for VaD patients. All controls had MMSE scores between 28 and 30. After the death of AD patients, the search for plaque with a brain autopsy was done to confirm the diagnosis of AD as the only definitive way.

### Serum Collection and Preparation

In this study, we recruited 404 AD subjects, 52 VaD patients, and 404 age- and gender-matched controls. Detailed demographic information of the subjects enrolled in this study is presented in Table 1. These patients were diagnosed clinically as having probable AD according to Diagnostic and Statistical Manual of Mental Disorders-IV (DSW-IV), International Classification of Diseases-10 (ICD-10), and NINCDS-ADRDA criteria, and they were clinically diagnosed as having probable VaD using NINDS-AIREN criteria. The Mini-Mental State Exam (MMSE) was used to assess the cognitive severity of dementia. Controls had an MMSE score between 28 and 30 without the cognitive decline, and they did not present a history of depression, psychosis, or use of medications that had side effects of cognitive impairments. All controls were followed clinically for 2 years in order to rule out the development of cognitive decline. In this study, no subject, originating from Northern Han Chinese populations, presented with major and known co-morbidities, including hypertension, cardiopathy, diabetes, or renal dysfunction. Written informed consent was acquired from all subjects, and the protocol was approved by the Institute Ethical Committee of Ludong University. Blood was collected in evacuated collection tubes without anticoagulant and allowed to clot for 2 h on ice prior to centrifugation at 4,000 g for 8 min at 4°C. After that, serum samples were aliquoted (50 ml/tube) and stored in −80°C.

**Table 1:** Demographics characteristics of the study samples.

	Control	AD	VaD	*p*-value
Number	404	404	52	N.S.
Age, years	74.7 ± 6.2	75.2 ± 5.5	75.1 ± 5.8	N.S.
Sex, M/F	204/200	199/205	25/27	N.S.
BMI	27.1 ± 4.8	26.9 ± 4.2	27.3 ± 4.6	N.S.
MMSE, points	28.7 ± 0.8	17.1 ± 4.0	16.8 ± 3.3	<0.01
Education, years	9.1 ± 3.5	9.2 ± 3.7	8.8 ± 4.1	N.S.

*Data are presented as means ± SD. Student’s* t* test. MMSE: AD vs. Control, *p* < 0.01; VaD vs. Control, *p* < 0.01. N.S., not significant different. AD, Alzheimer’s disease; BMI, body mass index; MMSE, mini-mental status examination*.

### AD Animal Models

In this study, AD animal models include APP/PS1 double transgenic mice and Aβ_25–35_ intracerebroventricular-injected rats. We purchased APP/PS1 double transgenic mice and age-matched wild-type (WT) mice from the Jackson Laboratory Company, and Memantine from Sigma-Aldrich. Male Wistar rats (3 months old, 220–250 g) were obtained from the Experimental Animal Center of Ludong University. 1 nM Aβ_25–35_ was injected into the lateral cerebral ventricle of these rats. These AD animal model mice (rats) were randomly divided into two groups of 8–10 mice each: vehicle model group and Memantine (30 mg/kg) group. The administration by oral gavage was started at 12 months old and lasted for 12 weeks. All the experiments were approved according to the institutional guidelines of the Experimental Animal Center of Ludong University.

### Western Blot Analysis

The brain tissues were homogenized thoroughly in a RIPA lysis buffer containing 150 mM NaCl, 50 mM Tris (pH 7.4), 1% NP40, 0.5% sodium deoxycholate, and 0.1% SDS. Then, the samples were centrifuged at 25,000 g at 4°C for 60 min, and the supernatants were collected and stored at –80°C until use. The serum samples from these subjects were also dissolved in the above buffer. Before that, protein concentration was measured with a BCA kit. Subsequently, western blot analysis was made according to the protocol previously published. All the samples were subjected to electrophoresis, transferred onto PVDF membranes, and incubated with the primary antibodies: rabbit anti-Alix (1:500, Cell Signaling Technology), mouse anti-β-actin (1:10,000, Sigma), and mouse anti-IgG (1:10,000, Abcam). Digital images were obtained with the LAS4000 FujiFilm imaging system (FujiFilm, Japan), and the densitometric analysis was made by Quantity-One software (Bio-Rad, United States).

### ELISA Analysis

Alix levels in serum were measured using a commercially available human Alix quantitative sandwich enzyme immunoassay (Uscnk, Wuhan, China). The standards and test samples were pipetted into 96-well plates pre-coated with anti-Alix antibody and incubated for 2 h at 37°C subsequently. After the removal of the liquid from each well, 100 ml of biotin-conjugated antibody specific for Alix was added and incubated for 1 h at 37°C. Then, they were mixed gently until the solution appeared uniform at room temperature, and this was then washed with wash buffer three times. After that, the avidin conjugated HRP was added and incubated for 1 h at 37°C. To remove any unbound avidin-enzyme reagent, the aspiration/wash process was repeated five times. TMB substrate was added and incubated for 15–30 min at 37°C. After that, the stop solution was added. The optical density was determined using an MQX200 microplate reader (Bio-Tek, United States) set to 450 nm. The serum level of Alix in the samples was interpolated from kit-specific standard curves generated using GraphPad Prism software. CV% was less than 8% in the intra-assay precision (Precision within an assay), and CV% was less than 10% in the inter-assay Precision (Precision between assays). For the assessment, three samples of known concentration were tested 20 times on one plate. The detection range of the ELISA kit is 47–3,000 pg/ml. The detection limit of human Alix is 11.7 pg/ml. It was determined the mean OD value of 20 replicates of the zero standard added by their three standard deviations. High sensitivity and specificity for the detection of human Alix were shown in this assay, and, moreover, no significant cross-reactivity or interference between human Alix and analogs was observed. Similarly, Aβ_40_ levels in serum were also measured using a commercially available human Aβ_40_ quantitative sandwich enzyme immunoassay (Abcam, Cambridge, UK).

### Statistical Analysis

The data were analyzed using SPSS 13.0 software. Comparison between the groups was made using Student’s *t*-test and one-way ANOVA with Tukey–Kramer method as a *post hoc* test. Correlations between Alix level and MMSE scores were performed with the Spearman correlation coefficient. The sensitivity and specificity of the measured variable for AD diagnosis were determined by ROC analysis. The best cut-off value was selected as those which minimize the sensitivity-specificity difference and maximize discriminating power of the tests. Statistical significance was set at *p* < 0.05.

## Results

### Decreased Alix Expression in AD Patients

**Figure 1:**
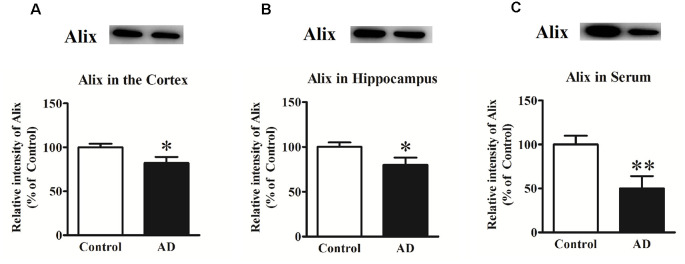
**(A)** Western blot analysis of Alix in the cortex tissues from Alzheimer’s disease (AD) patients and controls. **(B)** Western blot analysis of Alix in the hippocampus tissues from AD patients and controls. **(C)** Western blot analysis of Alix in serum samples from AD patients and controls. Quantified results were normalized to β-actin/IgG expression. Values were expressed as percentages compared to the controls (set to 100%) and represented as means ± SEM. *n* = 12 per group. *p < 0.05, **p < 0.01 vs. the control group, Student’s *t*-test.

In our previous study, Alix was identified to be significantly decreased in APP/PS1 transgenic mice compared to the age-matched WT mice as well as in serum samples from a small number of AD patients (Sun et al., 2015). To determine whether Alix was also downregulated in brain tissues of AD patients, the expression of Alix in the cortex and hippocampus tissues from AD patients and controls after death was also assessed. Western blot analysis showed a statistically significant decrease in protein expressions by 18% (Figure 1A) in the cortex and by 20% (Figure 1B) in the hippocampus of AD patients compared to the controls. Meanwhile, the serum level of Alix was validated again in our present study, and a significant decrease of 50% (Figure 1C) was observed in AD sera.

### Serum Alix and Aβ Levels Detected by ELISA

**Figure 2:**
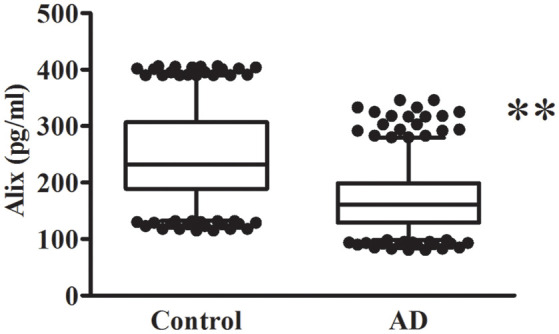
Alix levels in serum are presented as box plots for AD patients and healthy controls. The lower and upper sides of the boxes indicate the 25th and 75th percentiles, and the horizontal lines indicate the means. The lower and upper whiskers are also shown that indicate the minimum and maximum values, respectively. *n* = 404 per group. **p < 0.01 vs. the control group, Student’s *t*-test.

The decreased serum level of Alix was validated subsequently by ELISA in a larger population. All samples were matched for age, gender distribution, and education. MMSE score is an important measure of the cognitive level. Relative to the control group, the AD group had a lower MMSE score (mean MMSE score: 28.7 ± 0.8 vs. 17.1 ± 4.0; Table 1). As shown in Figure 2, the results of ELISA showed that Alix level was markedly lower in AD than that in the control group in serum (Control: 246.3 ± 77.4 pg/ml, AD: 168.9 ± 53.4 pg/ml, *p* < 0.01). The 95% confidence intervals (CIs) were 163.7–174.2 pg/ml in AD and 238.7–253.8 pg/ml in the control group in which no overlap between AD and Control was shown. Corresponding results are shown in Table 2. In Table 2, the Aβ_40_ level in serum was also shown. Aβ_40_ had a significantly increased expression in AD compared to the control (Control: 32.4 ± 8.8 pg/ml, AD: 41.0 ± 13.3 pg/ml, *p* < 0.01). The 95% confidence intervals (CIs) were 39.7–42.3 pg/ml in AD as well as 31.5–33.2 pg/ml in the controls.

**Table 2:** Alix and Aβ_40_ levels in serum of AD patients and Controls.

		Control	AD
Alix	Protein Level (pg/ml)	246.3 ± 77.4	168.9 ± 53.4**
	The 95% CIs	238.7–253.8	163.7–174.2
Aβ_40_	Protein Level (pg/ml)	32.4 ± 8.8	41.0 ± 13.3**
	The 95% CIs	31.5–33.2	39.7–42.3

**p-value < 0.01 in student’s *t* test of difference in means between AD and control group. Data are shown as means ± SD. *n* = 404. CIs: confidence intervals.

### Correlation Analysis

**Figure 3:**
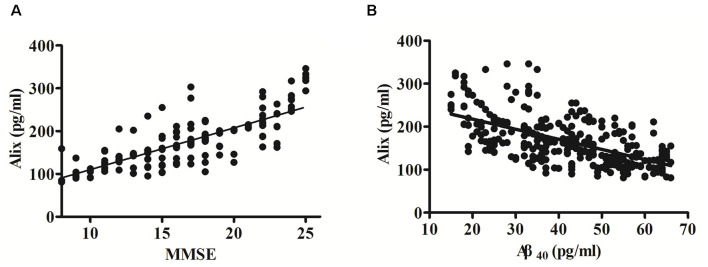
**(A)** Correlation analysis between Alix serum levels and MMSE scores in AD patients. **(B)** Correlation analysis between Alix and Aβ_40_ in serum levels. Correlation was assessed using the Spearman correlation coefficient. A significant positive correlation between serum Alix level and MMSE score (*r* = 0.80, *p* < 0.001) was observed, as well as a significant negative correlation between Alix and Aβ_40_ in serum levels (*r* = −0.60, *p* < 0.001). Correlation lines are also shown.

The potential correlation between the cognition (evaluated by MMSE scores) and the levels of serum Alix was analyzed and shown in Figure 3A. Spearman correlation analysis showed a significant positive correlation within the AD group (*r* = 0.80, *p* < 0.001). It is generally accepted that the risk of AD dementia is associated with Aβ. Previous studies have reported that Aβ levels are closely associated with incident AD risk (Reiss et al., 2018). In our present study, there was a significant increase of serum Aβ_40_ levels in AD patients compared to the controls, as seen in Table 2. Correlation analysis between serum Alix and Aβ_40_ levels was performed. As shown in Figure 3B, it revealed a very good negative correlation with statistically significant between serum Alix and Aβ_40_ levels (*r* = −0.60, *p* < 0.001), suggesting that Alix was probably involved in the amyloid pathogenesis of AD.

### ROC Analysis

**Figure 4:**
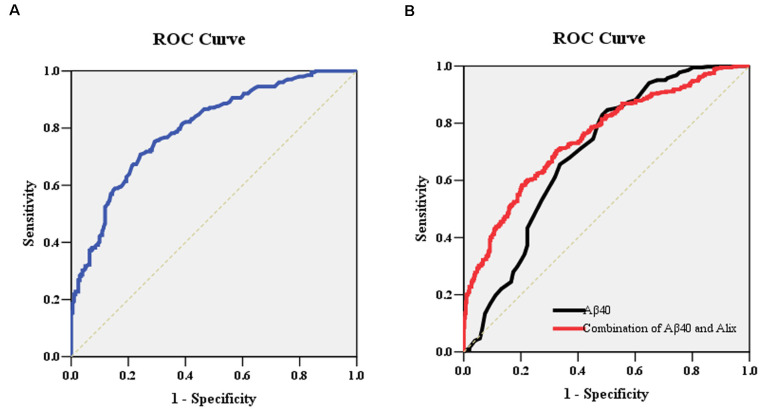
**(A)** ROC curve analysis for serum Alix concentration and the prediction of the presence of AD. The AUC was 0.80. The optimal cut-off value (199.5 pg/ml) was selected. The diagnostic accuracy for Aix protein levels was 74% with the sensitivity and specificity 76% and 71%, respectively. **(B)** ROC curve analysis for the combination of serum Aβ_40_ and Alix. The AUC value of Aβ_40_ was 0.69, and the AUC for the combined detection of Aβ_40_ and Alix was raised to 0.75. Abbreviations: ROC, receiver operating characteristic; AUC, area under the curve.

To determine the potential diagnostic value of Alix as a biomarker for discriminating between AD and healthy controls, the ELISA result of Alix was used to generate ROC curves (Figure 4A). The area under the curve (AUC) was determined to evaluate the diagnostic performance. The results showed that the AUC of Alix was 0.80, and the optimal cut-off point of 199.5 pg/ml was selected with the highest sum of sensitivity and specificity. The diagnostic accuracy for serum Alix was 74%, with 76% sensitivity and 71% specificity respectively, which could differentiate AD from controls. Aβ_40_ is known to be involved in the pathogenesis of AD and is currently one of the most promising biomarkers that might predict disease progression. Figure 4B showed the AUC value of Aβ_40_ was 0.69, and the AUC for the combined detection of Aβ_40_ and Alix was raised to 0.75.

### Decreased Serum Alix Levels in AD Compared to VaD

**Figure 5:**
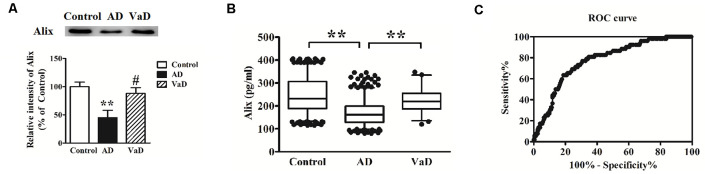
**(A)** Western blot analysis of Alix in brain tissues from AD patients, vascular dementia (VaD) patients and the controls. Equal volume of each sample was loaded and β-actin was used as a loading control. Values were expressed as percentages compared to control group (set to 100%), and represented as means ± SEM. *n* = 12 for AD and control group, and *n* = 8 for VaD group. **p < 0.01 vs. the control group, ^#^*p* < 0.05 vs. AD group. **(B)** Scatter plots of Alix levels in serum of VaD (*n* = 52), AD (*n* = 404) and Controls (*n* = 404) measured by ELISA. **p < 0.01. **(C)** ROC curve analysis for serum Alix between VaD and AD. The AUC was 0.777.

AD and VaD belong to the most common types of dementia. However, there are some difficulties to distinguish them by the clinical presentation and feature. In this study, Alix levels in AD and VaD sera were detected to estimate whether Alix could identify the two diseases as a serum biomarker. Western blot analysis showed that Alix was significantly decreased in brain tissues of AD patients compared to the controls, and had an obviously decreased level in AD compared to VaD, but there was no significant difference between VaD and the control groups (Figure 5A). ELISA analysis for serum samples showed a similar results to the western blot, and the serum level of Alix in VaD was detected to be 224.5 ± 55.7 pg/ml (Figure 5B). Through ROC curve analysis between AD and VaD, it was shown that the AUC of Alix was 0.777 (Figure 5C), suggesting that Alix might act as a marker to distinguish AD from VaD.

### Effect of Memantine on Alix Expression

**Figure 6:**
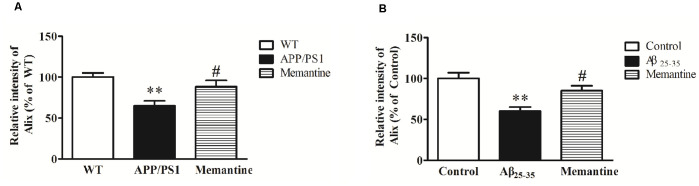
**(A)** Western blot analysis of Alix after the treatment of Memantine in serum samples from APP/PS1 mice and wild-type (WT) mice. Quantified results were normalized to IgG expression. Values were expressed as percentages compared to WT group (set to 100%), and represented as means ± SEM. *n* = 8–10. **p < 0.01 vs. the WT group, ^#^*p* < 0.05 vs. APP/PS1 mice. **(B)** Western blot analysis of Alix after the treatment of Memantine in serum samples from Aβ_25–35_ intracerebroventricular-injected rats and age-matched controls. Values were expressed as percentages compared to the control group (set to 100%), and represented as means ± SEM. *n* = 8–10. **p < 0.01 vs. the control group, ^#^*p* < 0.05 vs. Aβ_25–35_ intracerebroventricular-injected rats.

In order to observe the effects of positive anti-AD drugs on Alix expressions, APP/PS1 mice and Aβ_25–35_ intracerebroventricular-injected rats were orally administrated 30 mg/kg Memantine. Western blot analysis showed that Alix was significantly decreased in serum samples of AD animal models compared with the controls, and the decreases of Alix were significantly attenuated with the treatment of Memantine (Figure 6).

## Discussion

Cholesterol level in the central nervous system is known to be related to AD. Alix was reported to have a role to play in cholesterol homeostasis by facilitating the interaction between the E3-ubiquitin ligase NEDD4–1 (neural precursor cell-expressed developmentally downregulated gene 4) and its targets, ATP-Binding Cassette (ABC) transporters, including ABCG1 and ABCG4 (Alrosan et al., 2019). It was reported that ABCG1 could reduce the synthesis of Aβ peptides by enhancing cholesterol efflux from neurons to apolipoprotein E, and might play an additional proposed role in restricting the brain entry of Aβ in AD (Sano et al., 2016; Dodacki et al., 2017). ABCG4 is expressed mostly exclusively in astrocytes and neurons in the brain and could export cholesterol, oxysterols, and cholesterol synthesis intermediates (Kerr et al., 2011). Similar to ABCG1, ABCG4 was also found to be implicated in AD as a transporter of Aβ from cells (Dodacki et al., 2017). In addition, ABCG1 and ABCG4 could suppress γ-secretase activity and disturb γ-secretase distribution on the plasma membrane, leading to the decreased Aβ secretion, which may inhibit the development of AD (Sano et al., 2016). In our present study, a good negative correlation between serum Alix and Aβ_40_ levels was shown, which further proved that Alix was involved in the amyloid pathogenesis of AD.

Glutamate-induced neuronal cell death *via* N-methyl-D-aspartic acid receptors (NMDARs) excitotoxicity is thought to contribute to AD development (Zhang et al., 2016; Wang and Reddy, 2017). The influx of Ca^2+^ through NMDARs is essential for stimulating intracellular signaling cascades to cause cell death, but the precise molecular mechanisms of NMDARs in neuronal death still remain unclear (Hardingham and Bading, 2010; Szydlowska and Tymianski, 2010). Alix, a known modulator of caspase-dependent and caspase-independent cell death, has been found within the human postsynaptic density (PSD), in which NMDARs are central components and could trigger Ca^2+^-dependent neuronal cell death (Salim et al., 2019). Moreover, dopamine signaling is a critically important process in the brain, and dopamine receptors (DARs) are closely related to neurodegenerative diseases such as AD and have become an important target for the prevention and treatment of AD (Reeves et al., 2017; Pan et al., 2019). In a previous study, Alix was identified as a novel dopamine receptor-interacting protein, up-regulating DARs expression and playing important roles for their stability and trafficking (Zhan et al., 2008). Given that DARs also interact with NMDARs (Lee et al., 2002; Liu et al., 2006; Devor et al., 2017), we speculated that Alix might have the capacity to influence NMDARs triggered neuronal death. Furthermore, it was reported that the close proximity of both Alix and NMDARs allowed Alix to influence the downstream pathways following NMDARs activation in low or absent glutamate/glycine concentrations (Salim et al., 2019), and thus, it was speculated that Alix might act as a potential modulator of NMDARs function. Our present study showed that the down-regulation of Alix was significantly attenuated after the treatment with Memantine as an NMDA receptor antagonist in APP/PS1 mice and Aβ_25–35_ intracerebroventricular-injected rats, which further confirmed that Alix might remain closely tied to the NMDA-related pathology of AD.

One of the most important symptoms in AD patients is brain atrophy. A previous report showed that Alix knock-out mice suffer from the severe reduction of brain volume and size, especially in both mediolateral length and thickness of the cerebral cortex (Laporte et al., 2017). A previous report showed that overexpression of Alix in the chick neural tube induced massive apoptosis of neuroepithelial cells, leading to the reduction by 25% in the width of the neural epithelium (Mahul-Mellier et al., 2006). However, it is still unclear whether Alix is up-regulated or down-regulated in AD patients. Our present study confirmed that the expression of Alix was significantly decreased in postmortem brain tissues of AD patients. We speculate that the lack of Alix could induce apoptosis probably by disrupting the balance of the signal pathways in the apoptotic regulation.

In order to explore whether Alix could serve as an AD biomarker, we performed a series of experiments in our present study. Alix level was demonstrated to be significantly decreased in brain and serum samples of AD patients compared to the controls in our present study. The good correlation between MMSE scores and Alix levels suggests that Alix was closely related to disease severity of AD, and meanwhile, a good correlation between Alix and Aβ_40_ serum levels was also observed. ROC analysis showed that Alix had high diagnostic values as a reliable biomarker to distinguish patients with AD from the controls, as well as AD from VaD. Moreover the decreased expression of Alix was attenuated after the treatment of Memantine in APP/PS1 mice and Aβ_25–35_ intracerebroventricular-injected rats. The above results showed the large possibility of Alix as a potential indicator to predict the risk of AD and as a drug target to antagonize AD progression. It is known that Aβ could cause pore formation resulting in the leakage of ions and the disruption of cellular calcium balance, eventually promoting apoptosis, causing synaptic loss, and disrupting the cytoskeleton (Reiss et al., 2018), while Alix in combination with Ca^2+^ was demonstrated to be involved in the apoptotic process (Scheffer et al., 2014; Laporte et al., 2017). Thus, we speculate that the dysregulation of Alix probably plays an important role in the pathology induced by Aβ. Further investigation is needed to explore the exact roles of Alix in the pathogenesis of AD.

## Data Availability Statement

The original contributions presented in the study are included in the article, further inquiries can be directed to the corresponding author.

## Ethics Statement

The studies involving human participants were reviewed and approved by the ethics Committee on Human Experimentation of Ludong university. The patients/participants provided their written informed consent to participate in this study. The animal study was reviewed and approved by the ethics committee of Ludong university.

## Author Contributions

YS conceived and designed the studies. JH, JY, GC, and HG enrolled all the subjects and collected the serum samples. YS, JY, and JL performed the research. YS analyzed the data and wrote the manuscript. All authors contributed to the article and approved the submitted version.

## Conflict of Interest

The authors declare that the research was conducted in the absence of any commercial or financial relationships that could be construed as a potential conflict of interest.
